# Multi-Omics and Molecular Docking Studies on Caffeine for Its Skin Rejuvenating Potentials

**DOI:** 10.3390/ph18081239

**Published:** 2025-08-21

**Authors:** Peng Shu, Nan Zhao, Qi Zhou, Yuan Wang, Lanyue Zhang

**Affiliations:** 1 HBN Research Institute and Biological Laboratory, Shenzhen Hujia Technology Co., Ltd., Shenzhen 518000, China; 2 School of Biomedical and Pharmaceutical Sciences, Guangdong University of Technology, Guangzhou 510006, China

**Keywords:** caffeine, UV-induced skin damage, skin healing, anti-photoaging, ferroptosis pathway

## Abstract

**Background**: Caffeine (CA) exhibits promising reparative effects against UV-induced skin aging, but the specific mechanisms, including differences in gene and metabolite regulation and the involvement of signaling pathways, are still insufficiently elucidated. **Methods**: This study is on the repairing capability of CA to ultraviolet (UV)-induced skin aging and explores the ferroptosis pathway through in vitro cell experiments, a UV-aged mouse skin model, and molecular docking. **Results**: CA enhanced the vitality and proliferation of HaCaT cells, delayed cell aging, reduced reactive oxygen species levels, increased mitochondrial membrane potential, and activated the peroxisome proliferator-activated receptor pathway, as well as repaired UVB-induced cytoskeletal disorders. Simultaneously, CA reduced other related but undesirable biological mechanisms. Moreover, multi-omics and network pharmacology studies suggested that CA mitigated aging by modulating related metabolic and ferroptosis pathways. Additionally, CA effectively reduced lipid peroxidation and intracellular ferrous ion levels and regulated the expression of key ferroptosis proteins, and its potential anti-aging effects were also confirmed through the modulation of ferroptosis pathways. In addition, molecular docking revealed strong interactions between CA and related key proteins, further supporting the potentiality of CA. **Conclusions**: This study elucidates the effectiveness and potential mechanism of CA to reduce the UV-induced skin aging.

## 1. Introduction

It has been shown that excessive UV radiation is the main factor that causes human skin aging [1,2,3]. UV radiation often produces reactive oxygen species (ROS); degrades cellular lipid membranes, extracellular matrix, collagen, elastin fibers, and nucleic acids; activates apoptotic proteins; and thus leads to skin photoaging and even cancer [4,5,6,7]. In particular, UV radiation downregulates genes related to iron metabolism, thus affecting some key molecules in ferroptosis such as glutathione peroxidase 4 (GPX4) [8]. UV radiation activates matrix metalloproteinases (MMPs) and potentially disrupts iron ion distribution [9]. Therefore, UV radiation-induced skin aging is complex but seriously affects human health. Recently, natural bioactive compounds are in great demand for skin protection due to their outstanding bioactivity and safety. Among them, caffeine (CA), a natural methylxanthine alkaloid found in plants, has been increasingly used in skin protection because it can scavenge free radicals, reduce oxidative stress, decrease skin roughness and rhytides, inhibit photoaging-related enzymes, and thus prevent and repair skin senescence [10,11,12,13,14,15,16]. To improve CA skin permeation, a novel CA-loaded nanocarrier is developed as a nano-cosmeceutical tool against skin photoaging [17]. Some studies have indicated the good repair efficacy of CA in mitigating UV-induced skin aging, but the specific mechanisms, including differences in gene and metabolite regulation and the involvement of signaling pathways, are still insufficiently elucidated.

To elucidate the underlying mechanisms, particularly ferroptosis, this study employs multi-faceted experimental approaches to study CA repairing efficiency on UV-induced skin aging. In vitro UV-aged HaCaT cell experiments are used to assess its effects on cellular senescence, proliferation, and ferroptosis-related markers. Cell viability, cell migration, cytoskeleton, β-galactosidase activity, ROS, peroxisome proliferator-activated receptor alpha (PPAR-α), mitochondrial membrane potential detection, Fe^2+^ level, lipid peroxide, and western blotting are used to evaluate the changes in cellular behavior and expression. A mouse skin photoaging model induced by UV radiation is established. Skin appearance and pathological studies are assessed, including hematoxylin and eosin (HE), Masson, and Toluidine blue (TB) staining. Immunohistochemical staining is used to evaluate inflammatory factors and collagen fibers. The expression levels of malondialdehyde (MDA), superoxide dismutase (SOD), and hydroxyproline (HYP) are investigated by enzyme-linked immunosorbent assay (ELISA). In addition, transcriptomic, metabolomic, and network pharmacological analyses are combined to explore molecular pathways. Molecular docking is conducted to examine the interactions between CA and key proteins, including tumor necrosis factor-α (TNF-α), interleukin-1 β (IL-1β), interleukin-6 (IL-6), collagen type I (COL-I), nicotinamide adenine dinucleotide phosphate (NADPH), and PPAR-α. These integrated approaches aim to elucidate how CA modulates the expression of the senescence-associated secretory phenotype (SASP) to further orchestrate cellular repair processes, providing a foundation for understanding its potential as an anti-photoaging agent.

## 2. Results

### 2.1. Effects of CA on UV-Aged HaCaT Cells

**Figure 1:**
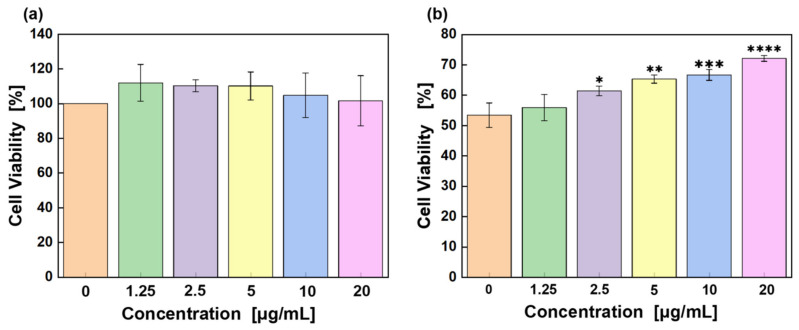
HaCaT cell viability after adding different concentrations of CA: (**a**) without UV radiation and (**b**) with UV radiation. Significant differences between the experimental groups (*n* = 3; * *p* < 0.05, ** *p* < 0.01, *** *p* < 0.001, and **** *p* < 0.0001, compared with the 0 μg/mL group).

The effect of CA on HaCaT cell survival is investigated by MTT assay (Figure 1a). The results indicate that cell viability is first increased and then decreased with increasing amounts of CA, suggesting that moderate CA is beneficial for HaCaT cell proliferation. However, excessive UV radiation usually causes serious damage to cells. Figure 1b shows that cell viability drastically decreases to about 50% after UV radiation. With the incorporation of increasing amounts of CA, cell viability gradually increases and reaches approximately 70% at a concentration of 20 μg/mL. It implies that CA can alleviate cellular damage induced by UV radiation and thus enhance cell growth.

**Figure 2:**
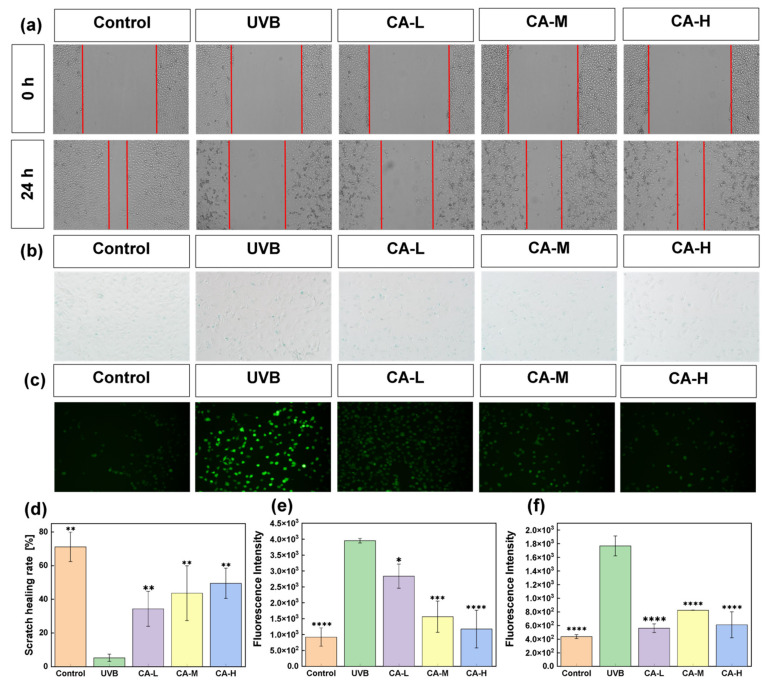
Repairing effects of CA on UV-damaged HaCaT cells: (**a**) scratch migration images, (**b**) β-galactosidase clearance, (**c**) immunofluorescence of ROS production with CA treatment, (**d**) cell migration rate, (**e**) the content of β-galactosidase, and (**f**) ROS levels. (*n* = 3; * *p* < 0.05, ** *p* < 0.05, *** *p* < 0.001, and **** *p* < 0.0001 indicated significant differences compared with the UVB group).

The cell scratch assay is a popular method for estimating cell migration and repair capacity, including the anti-photoaging effect of drugs in vitro [18]. From Figure 2a, UV radiation reduces cell migration, but it is significantly improved by more CA. After 24 h, HaCaT cells in the CA-H group exhibit the best proliferation effect, with a migration rate of 50% (Figure 2d). This suggests that CA has potential skin damage repair effects, with a significantly increased migration rate at higher concentrations. During cell senescence, the bioactivity and expression level of β-galactosidase increase, making it a common marker for cell aging [19]. Figure 2b shows that compared to the UVB group, the blue stains resulting from β-galactosidase are more prominent after UV radiation, but they are significantly reduced by CA treatment. Figure 2e reveals that the average optical density is significantly increased by UV radiation, while it is greatly reduced with more CA. On the other side, ROS plays a crucial role in cellular signal transduction, and increased ROS levels often accompany ferroptosis. Low concentrations of ROS function as “redox messengers” involved in intracellular signal transmission and regulation, while high concentrations of ROS disrupt redox balance, affecting normal signal transduction and gene expression [20]. In Figure 2c, green ROS fluorescence becomes visible after UV radiation but is significantly reduced after CA treatment. Figure 2f indicates that the average optical density in the UVB group is significantly increased, whereas its level is effectively inhibited by CA treatment (*p* < 0.0001).

**Figure 3:**
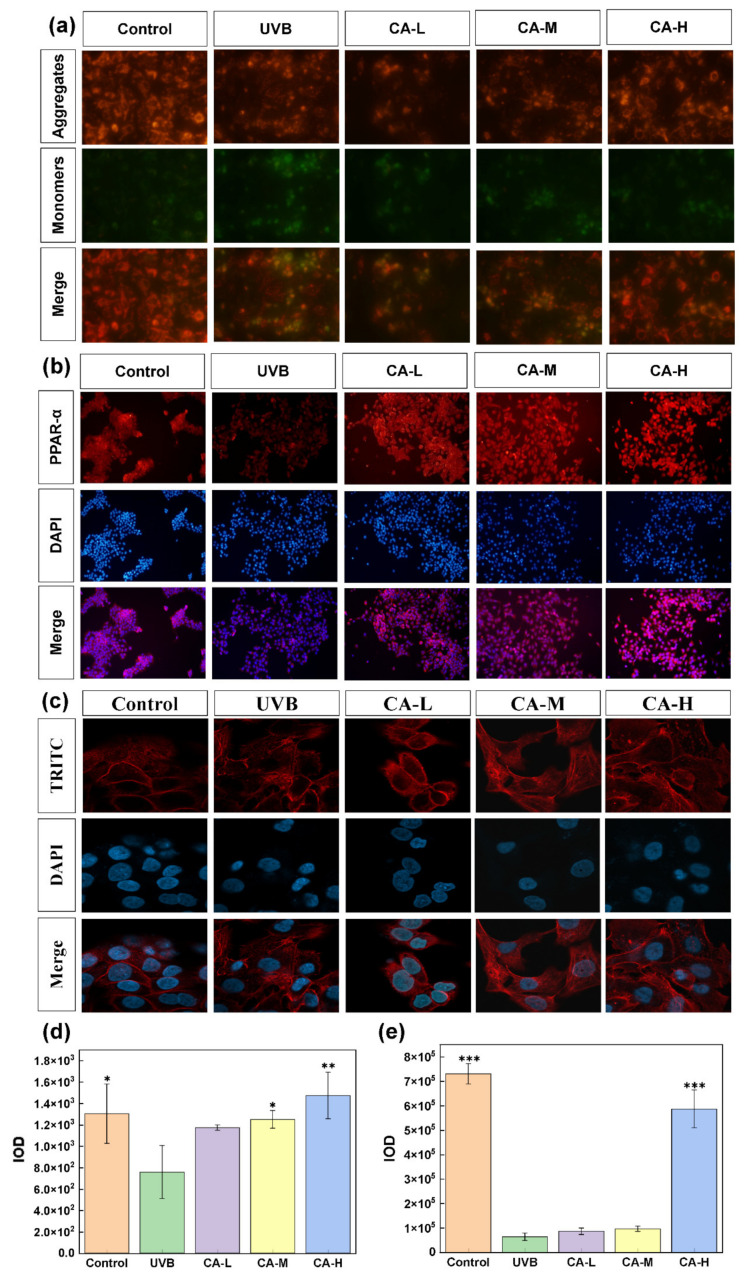
Effects of CA on mitochondrial membrane potential in HaCaT cells: (**a**) cellular mitochondrial membrane potential, (**b**) fluorescence expression of PPAR-α, (**c**) HaCaT cytoskeleton images under laser confocal microscopy, (**d**) quantitative analysis of JC-1 aggregates, and (**e**) fluorescence intensity of PPAR-α. (*n* = 3; * *p* < 0.05, ** *p* < 0.01, *** *p* < 0.001 indicated significant differences compared with the UVB group).

Moreover, mitochondria are essential organelles in cell metabolism, determining whether all the functional activities of the cell can proceed normally. Mitochondrial membrane potential is one of the key indicators of mitochondrial activity and cellular vitality, and its reduction is considered a hallmark event in the early stages of cell apoptosis [21]. Figure 3a shows that compared to the control group, the UV group exhibits stronger green fluorescence and weaker red fluorescence, indicating a decrease in mitochondrial membrane potential (*p* < 0.05) and an early apoptotic signal. The CA treatment groups with different concentrations show a gradual increase in red and a decrease in green fluorescence, indicating the gradual recovery of mitochondrial membrane potential. From Figure 3d, the high-concentration CA treatment group shows a significant increase (*p* < 0.01). The results indicate that CA has a specific restorative effect on mitochondrial membrane potential, thereby improving cellular vitality and protecting cellular integrity.

UV radiation can trigger skin inflammatory responses, releasing various inflammatory mediators and accelerating skin aging. Once activated, PPAR-α can inhibit inflammatory signaling pathways, reduce the expression and release of inflammatory factors such as TNF-α and IL-1β, alleviate inflammation damage to skin cells, and exert an anti-photoaging effect [22]. Moreover, along with the activation of PPAR-α, the activation of nuclear factor-κB (NF-κB) is inhibited, thereby reducing the expression of genes related to photoaging, such as inflammatory factors and MMPs [23,24,25,26,27]. Excessive UV exposure accelerates skin inflammation and aging [28,29,30,31]. To explore the mechanism of CA on anti-inflammation and determine whether CA can regulate the expression of related proteins (NF-κB) by activating the PPAR-α signaling pathway, the related PPAR-α signaling pathway is analyzed at the protein level and nuclear localization. Figure 3b shows that fluorescence in the UV group is weakened compared to the control group. At the same time, it almost recovers to the level of the control group after being treated with different concentrations of CA. Among them, the recovery of the CA-H group is significant (Figure 3e). These results suggest that CA can antagonize skin photoaging by activating the intracellular PPAR-α signaling pathway, regulating NF-κB levels, and decreasing cell inflammation [32].

The cytoskeleton, consisting of three main types of protein filaments—microtubules, actin filaments, and intermediate filaments—plays a vital role in maintaining cell shape and internal organization, affecting various cellular functions such as movement, material transport, energy conversion, signal transduction, and differentiation [33]. In Figure 3c, the cytoskeleton of the control group is regular and filamentous. In the UVB group, the actin cytoskeleton, as visualized by phalloidin staining, shows a disordered structure in damaged cells. After treatment with different concentrations of CA, the actin cytoskeleton returns to a more normal filamentous state, where the CA-H group shows the most effective repair effect. These findings suggest that CA can repair cell damage caused by inflammatory responses.

### 2.2. Repairing Effect of CA on UV-Induced Aged Mice Skin

**Figure 4:**
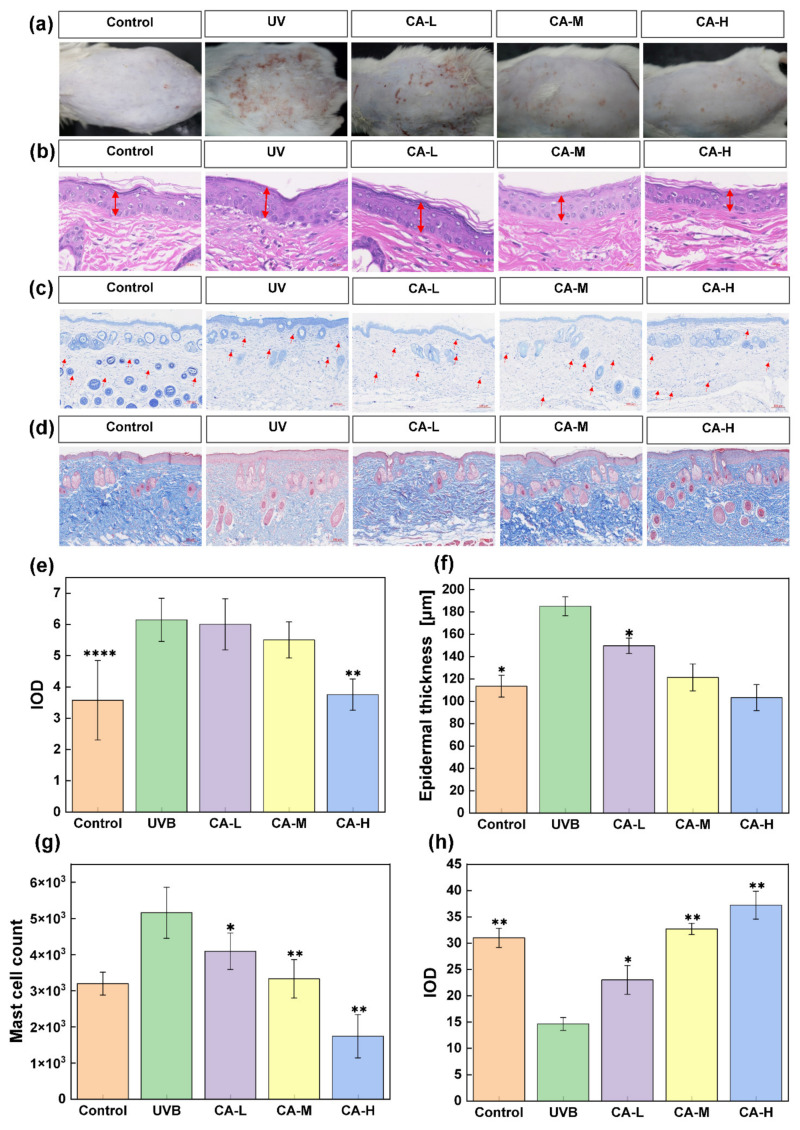
Skin appearance and tissue section staining of photoaged mice skin: (**a**) erythema and wrinkles in the dorsal skin, (**b**) HE stains the back epidermis (scale bar = 20 μm; The red arrow indicates the thickness of the epidermis), (**c**) toluidine blue staining of the skin (scale bar = 100 μm; The red arrow indicates the expression of the protein), (**d**) Masson staining of the skin (scale bar = 100 μm). Statistical analysis of (**e**) visual scores recorded on the dorsal skin, (**f**) representative epidermal thickness, (**g**) mast cell content, and (**h**) collagen staining content. Significant differences existed between the experimental groups (*n* = 6; * *p* < 0.05, ** *p* < 0.01, **** *p* < 0.0001, indicating significant differences compared with the UV group).

UV-induced skin lesions always cause abnormalities such as increased skin surface roughness, abnormal epidermal hyperplasia, and abnormal epidermal thickness [34]. From Figure 4a, compared with the control group, there is a significant increase in wrinkles and erythema on the depilated backs of the model group after UV radiation. After treatment with CA, skin surface roughness and skin thickness are significantly reduced. Compared to the control group, the UV model group exhibits a significant increase in skin thickness (*p* < 0.01) (Figure 4e). Notably, the CA-H group shows the most significant effect in mitigating this trend compared to the model group.

Moreover, HE staining in Figure 4b indicates that the epidermis thickness is standard in the control group, while the UV model group exhibits a significantly thicker stratum corneum. CA treatment alleviates the above skin problems. Figure 4f shows that the CA-L, CA-M, and CA-H groups can somewhat relieve abnormal epidermal proliferation, with the CA-H group having the most significant effect [35,36]. Abnormal mast cell proliferation is a key feature of skin inflammation [37,38], and skin inflammation accelerates the progress of skin photoaging [39]. Blue toluidine staining is a quick and easy way to analyze whether mast cells proliferate abnormally, which is used to assess the effect of CA on abnormal mast cell proliferation (Figure 4c). The UV group shows a significantly abnormal increase in dermal mast cells. Mast cell infiltration in the CA group is decreased at different concentrations. CA concentration is inversely related to mast cell infiltration levels, and this becomes more apparent with increased concentration, indicating that CA may remit the abnormal increase in mast cell numbers caused by inflammation (Figure 4g). UV has a strong penetrating power and can directly penetrate the epidermis and destroy collagen fibers within skin tissues. Therefore, the content of collagen fibers in skin tissues can be used as a common indicator to evaluate the effectiveness of CA against photodamage. Masson staining is widely used to observe tissue structure and cell morphology, where stained collagen fibers are blue and muscle fibers are red. In the control group, collagen fibers are tightly and neatly arranged, with a uniform blue color, signifying a healthy and intact collagen network. However, there is a notable reduction in collagen fibers, with less prominent blue staining and a disorganized arrangement in the UV group (Figure 4d). The CA treatment groups (CA-L, CA-M, and CA-H) show remarkable improvements. Compared to the model group, the number of collagen fibers in the CA-treated groups increases significantly, as evidenced by enhanced blue staining. Integrated optical density values, which are quantitatively measured from the stained area, also show a significant increase after CA treatment (Figure 4h). These findings demonstrate that the local application of CA is pivotal in effectively repairing the damage to collagen fibers caused by UV radiation. CA helps restore the amount of collagen and promotes a more organized and healthy arrangement of collagen fibers, highlighting its potent collagen-protecting and repairing abilities against UV-induced skin damage.

**Figure 5:**
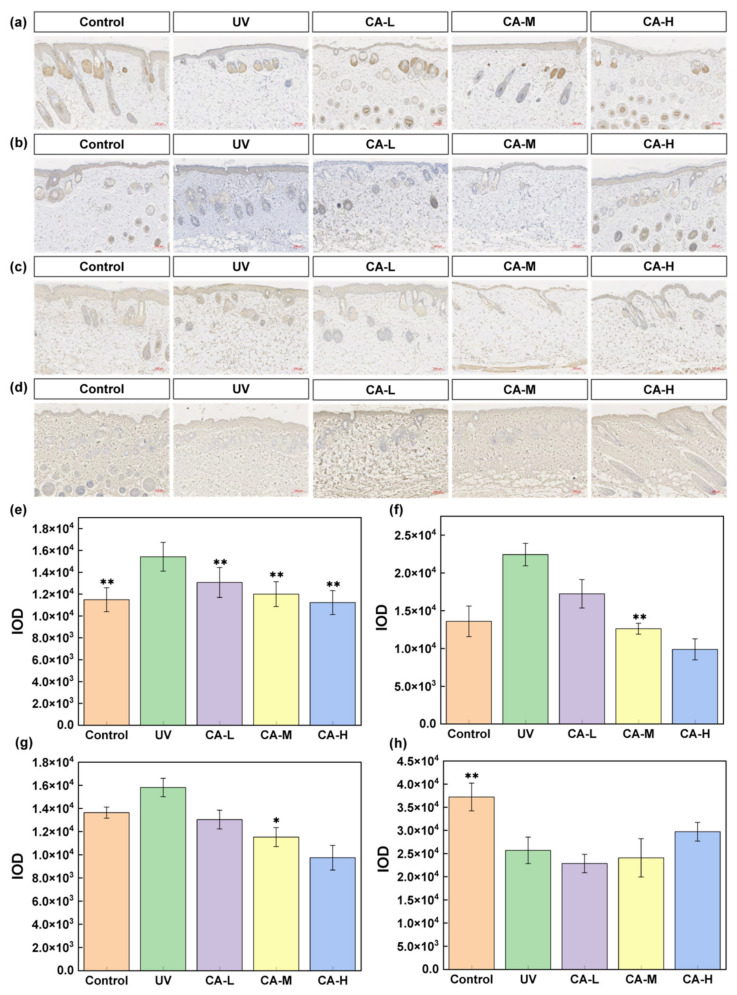
Immunohistochemistry images and analysis: (**a**) TNF-α, (**b**) IL-1β, (**c**) IL-6, and (**d**) Col-I staining images (scale bar = 100 μm); (**e**) TNF-α, (**f**) IL-1β, (**g**) IL-6, and (**h**) Col-I expression statistical analysis. (*n* = 6; * *p* < 0.05, and ** *p* < 0.01 indicated significant differences compared with the UV group).

Excessive UV radiation increases proinflammatory cytokines in the skin and thus accelerates skin aging. The key cytokines involved in the pathogenesis of skin aging included interleukins such as IL-6, COL-I, TNF-α, and IL-1β. The expression levels of IL-6, TNF-α, and IL-1β are typically increased, while they are reduced for COL-I during aging [40]. Immunohistochemical analysis reveals significantly higher expression of IL-1β, IL-6, and TNF-α in the UV model group, along with markedly lower expression of COL-I, confirming the successful construction of the experimental model (Figure 5). After treatment with different concentrations of CA, the expression of IL-1β, IL-6, and TNF-α is much lower than in the UV model group. The effect of the CA-H group on IL-1β is more significant, that of the CA-L group on IL-6 is more significant, and that of the CA-M group on TNF-α is more significant. After CA-H treatment, COL-I expression is higher than that of the model (UV) group. Obviously, CA can alleviate the adverse effects of UV-induced skin photoaging by inhibiting the production of IL-1β, IL-6, and TNF-α and upregulating the expression of COL-I.

The antioxidant enzyme, SOD, can neutralize superoxide radicals, maintain oxidative balance, and shield skin from UV-induced damage, inflammation, and erythema. HYP, predominant in collagen, bolsters collagen stability and repair as a biomarker for connective tissue degradation. MDA levels indicate lipid peroxidation and tissue oxidative damage, reflecting antioxidant capacity [41]. SOD and HYP in the brain tissue of the model (UV) group exhibit a decreasing trend, while MDA content demonstrates an increasing trend (Table 1). Compared with the model group, SOD content in the CA-L group is increased by 31.17 U·Mgprot^−1^. Conversely, HYP and MDA content decrease by 0.72 and 0.8 U·Mgprot^−1^, respectively. Table 1 further indicates that as CA dosage increases, SOD levels increase, while HYP and MDA levels decrease. This shows that different concentrations of CA exhibit a favorable recovery effect on changes induced by UV exposure, and the degree of recovery is positively correlated with CA concentration. This suggests that CA may play a role in anti-photoaging by modulating enzyme content in the skin.

### 2.3. Regulating Ferroptosis in HaCaT Cells

**Figure 6:**
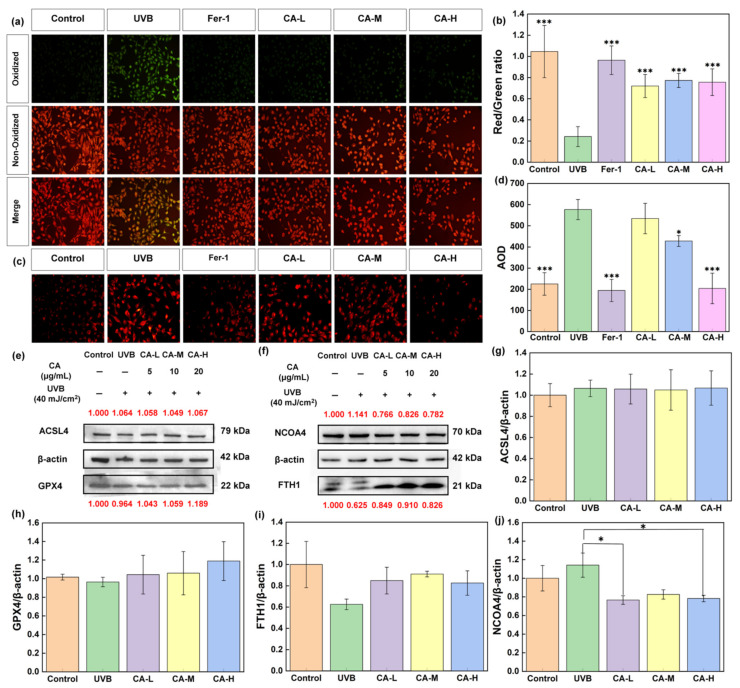
(**a**,**c**) Amounts of lipid peroxides in HaCaT cells: (**b**,**d**) effect of CA on Fe^2+^ level in photoaged HaCaT cells; and (**e**–**j**) effects of CA on the expression of ACSL4, GPX4, FTH1, and NCOA4. (*n* = 3; * *p* < 0.05 and *** *p* < 0.001 indicated significant differences compared with the UVB group).

Under the action of ferrous iron (Fe^2+^) or ester oxygenase, unsaturated fatty acids in the cell membrane undergo lipid peroxidation to produce a large amount of lipid peroxides (LPOs), thereby inducing cell death. The level of lipid peroxidation in cells is thus an important indicator for evaluating ferroptosis [42]. The lipid peroxidation fluorescent probe (C11 BODIPY581/591) is highly selective and sensitive to LPO. Under normal conditions, it exhibits a reduced state (red fluorescence), while its fluorescence characteristics change to enhanced green fluorescence after reacting with LPO. The increase in the green/red fluorescence ratio indicates an increase in intracellular LPO [43]. From Figure 6a, compared with the control group, the green fluorescence level is increased after UV radiation, while it is reduced by CA treatment. In Figure 6b, the green/red ratio is increased for the UVB group, indicating that the LPO level is increased. It decreases after ferrostatin-1 and CA treatment, indicating that its level significantly reduces after ferrostatin-1 and CA treatment (*p* < 0.001). However, Fe^2+^ is an essential factor in ferroptosis, which can generate potent oxidizing free radicals (such as hydroxyl radicals) through the Fenton reaction. Free radicals can trigger peroxidation of intracellular lipids, damage cell membranes, and lead to cell death. FerroOrange can specifically detect free Fe^2+^, and irreversibly generate an orange fluorescent product after reaction, where its fluorescence intensity is proportional to the content of Fe^2+^ [44]. From Figure 6c,d, the Fe^2+^ level is increased after UVB radiation. After treatment with ferrostatin-1, its level is significantly reduced (*p* < 0.001). The Fe^2+^ levels are also downregulated by different concentrations of CA, of which the high concentration group is significant (*p* < 0.001). These results indicate that CA can downregulate superoxide and Fe^2+^ levels in HaCaT cells induced by UVB, thereby inhibiting cell ferroptosis.

To further investigate the anti-aging effects of CA, western blot analysis is used to quantify expression levels of key proteins (Figure 6e,f), acyl-CoA synthetase long-chain family member 4 (ACSL4), GPX4, ferritin heavy chain 1 (FTH1), and nuclear receptor coactivator 4 (NCOA4). ACSL4, a long-chain fatty acyl-CoA synthetase [45], and GPX4, a crucial antioxidant enzyme, are well-established regulators of ferroptosis [46], with ACSL4 promoting and GPX4 inhibiting the process. FTH1, a ferritin heavy chain, and NCOA4, a nuclear receptor coactivator, are implicated in iron homeostasis and ferroptosis, with NCOA4 mediating ferritinophagy, a process that releases iron and promotes ferroptosis [47]. Investigating these proteins is to understand how CA treatment affects the balance between pro-ferroptotic and anti-ferroptotic factors, thereby influencing cellular senescence. From Figure 6g–j, the expression of ACSL4 decreases after treatment with low and medium concentrations of CA compared to the UVB group, but the difference is subtle. The expression of GPX4 exhibits a concentration-dependent increase in CA treatment. FTH1 expression is notably improved by CA treatment, with the most beneficial effects observed at moderate doses, which may suggest an adaptive response to iron metabolism. Furthermore, significant reductions in NCOA4 levels are observed in the CA-L and CA-H groups compared to the UVB group (*p* < 0.05), implying a decrease in ferritinophagy and potentially less iron-induced oxidative stress. These findings indicate that CA treatment can modulate the expression of ferroptosis-related proteins and cellular aging, suggesting its potential as an anti-aging agent by affecting the ferroptosis pathway.

### 2.4. Skin Transcriptome Analysis

**Figure 7:**
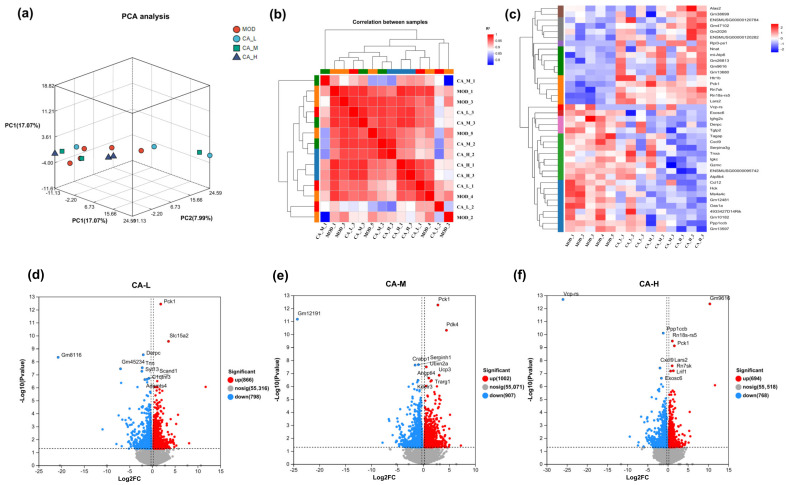
(**a**) PCA diagram, (**b**) sample correlation, (**c**) cluster heat map, and (**d**–**f**) volcanic plots of gene expression levels of the model group and CA-L, CA-M, and CA-H, respectively (*p* < 0.05).

The results showed that the Clean Data (quality-controlled data) of each sample exceeded 5.75 Gb, the error rate (average base error rate of the quality-controlled sequencing data) was lower than 0.0236%, Q20 (the proportion of bases with sequencing quality above 99%) was higher than 97.57%, and Q30 (the proportion of bases with sequencing quality above 99.9%) was higher than 95.38%. These indicators demonstrate that the quality of the sample data in this study is extremely high. After data quality control, the Clean Data were aligned with the reference genome, and the alignment rate of each sample was found to be higher than 96.86%, indicating high gene expression levels and that the data are suitable for subsequent analysis. Principal component analysis (PCA) is used to assess genetic variations and similarities within each group. Correlation analysis among samples reveals normal distributions, with no outliers identified in Figure 7a. Pearson’s correlation coefficient (R^2^) is used as the metric to evaluate biological replication correlation during sample correlation analysis. From Figure 7b, each sample exhibits an absolute value of R^2^ closer to 1, indicating a strong correlation between duplicate samples. In Figure 7c, genes that exhibit significant expression in the model group show reduced expression in the CA-M group. The cluster heatmap visualizes sample clustering based on differential gene expression. The horizontal axis displays sample information and hierarchical clustering results, whereas the vertical axis represents the differential genes and their corresponding clusters. High expression levels are shown in red, and low expression levels are shown in blue. CA primarily targets the upregulation of genes such as Alas2, Gm38699, ENSMUSG00000120784, Gm47102, Gm2026, ENSMUSG00000120282, Rpl3-ps1, Nnat, mt-Atp6, Gm26813, Gm9616, Gm13680, Htr1b, Pck1, Rn7sk, Rn18s-rs5, and Lars2. Conversely, it downregulates the expression of genes, including Vcp-rs, Exosc6, Ighg2c, Derpc, Tgtp2, Tagap, Cxcl9, Serpina3g, Tnxa, Igkc, Gzmc, ENSMUSG00000095742, Atp8b4, Ccl12, Hck, Ms4a4c, Gm12481, Oasia, 4933427D14Rik, Gm10182, Ppp1ccb, and Gm13597. Furthermore, many genes associated with ferroptosis are regulated, including Alas2, Htr1b, Pck1, Lars2, Cxcl9, and Ccl12.

Criteria for defining genetic screening differences include *p* < 0.05 and |log_2_ FC| > 1.2. The volcanic plot illustrates the total number of genes identified in the differential groups, highlighting significantly upregulated genes. In the volcano plot, the horizontal axis depicts changes in gene expression ratios, while the vertical axis reflects the statistical significance of gene expression. Red dots represent upregulated genes, blue dots represent downregulated genes, and gray dots indicate non-differential genes. From Figure 7d–f, the CA-M group exhibits the most pronounced differences in upregulated genes. In addition, phosphoenolpyruvate carboxykinase 1 (PCK1) is identified as a significant upregulated gene common to all three groups. PCK1 functions as a rate-limiting enzyme during the first step of the gluconeogenesis pathway, supplying cells with additional energy substrates such as glucose to support daily cellular metabolic demands. This helps to alleviate the cellular aging caused by insufficient energy supply. Now, it has been shown that PCK1 can clear ROS [48], which may reduce oxidative stress levels in senescent cells.

Genes associated with senescent cells are listed in Table 2. Ccl (chemokines) are small proteins in the cytokine family, primarily involved in directing white blood cells and other immune cells towards specific tissues or organs. Studies have shown that mRNA expression of Ccl is upregulated in older women [49], while observed downregulation suggests that CA may have a role in reversing senescent cells. Matrix metalloproteinase 12 (MMP12) is an enzymatic protein involved in the extracellular matrix breakdown. Usually, MMPs and tissue inhibitors of metalloproteinases (TIMPs) interact synergistically to maintain skin collagen balance. MMP shows a higher level of aging skin and a significantly lower level after CA treatment.

**Figure 8:**
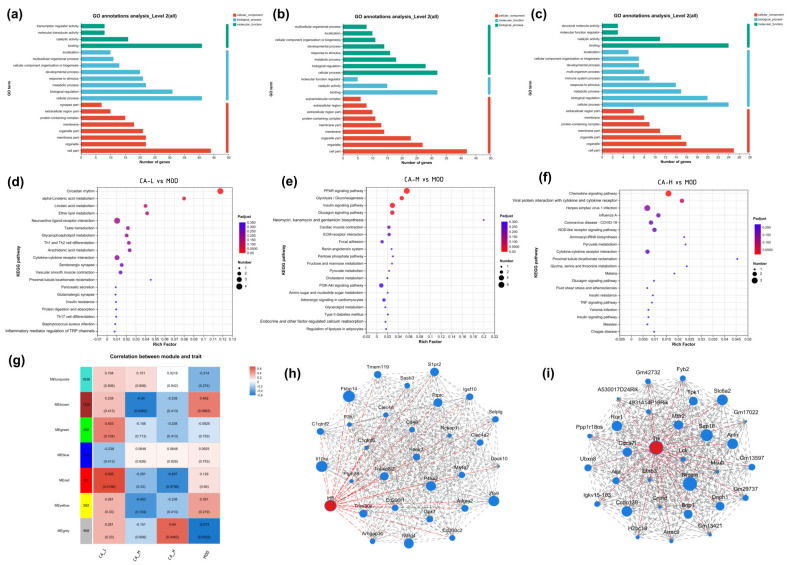
(**a**–**c**) GO enrichment maps of the model group with CA-L, CA-M, and CA-H, respectively; (**d**–**f**) KEGG enrichment maps of the model group with CA-L, CA-M, and CA-H, respectively; (**g**) module-feature association analysis; and (**h**) visualization of the key gene network of the brown module and (**i**) red module. The red dots are the genes with the highest correlation.

Gene ontology (GO) is a globally recognized framework for classifying gene functions, divided into three main categories: molecular function (MF), biological process (BP), and cellular component (CC). Herein, the ordinate is the secondary classification of GO, while the abscissa is gene expression levels. Kyoto Encyclopedia of Genes and Genomes (KEGG) can be used to determine which differentially expressed genes (DEGs) are enriched in and the relationship between up- and down-regulation within these pathways. From Figure 8a–c, GO gene enrichment is primarily observed in the categories of binding, cell part, and cellular process across the three sample groups. The identified pathways are not only abundant but also significantly enriched.

KEGG analysis reveals the enrichment of 20 signaling pathways in each comparison group, primarily associated with metabolism, cytokine activity, inflammatory responses, and other related pathways. The three compound groups are likely to regulate diverse signaling pathways through multiple target proteins, including COL-I, IL-6, IL-1β, etc. From Figure 8d–f, there is notable enrichment in these specific pathways. Specifically, the MOD vs. CA-L group is enriched in circadian rhythm and the α-linolenic acid metabolism signaling pathway. The MOD vs. CA-M group is enriched in PPAR and glycolysis/gluconeogenesis pathways. Enrichment analysis of MOD vs. CA-H groups reveals significant involvement in the chemokine pathway and protein interactions with cytokines and cytokine receptors, as shown in Figure 8d–f. It is worth mentioning that the three groups have similar pathways, possibly due to their identical treatment mechanisms for skin photoaging. Genes that are significantly regulated in all KEGG pathways are listed in Table 3. The PPAR pathway is a crucial cellular signaling pathway involving the activation and regulation of the PPAR protein family, which are nuclear receptors that play key roles in regulating lipid metabolism, glucose metabolism, and inflammatory responses. It has been demonstrated that PPAR-α can mitigate ferroptosis and mitochondrial damage induced by myocardial ischemia/reperfusion injury by regulating 14-3-3η [50]. PI3K-Akt is another important signaling pathway that involves activating and regulating key proteins such as phosphoinositide 3-kinase (PI3K) and protein kinase B (Akt). This pathway plays an important role in various physiological processes, including cell growth, survival, proliferation, and metabolic regulation. Studies have shown that curcumin [51] treatment significantly restored phosphorylation levels of PI3K, AKT, and cyclic AMP-dependent reactive element binding protein (CREB) proteins that are inhibited by RAS-selective lethal 3 (RSL3), thus demonstrating its anti-ferroptosis effect [52]. TNF (tumor necrosis factor) is a critical cellular signaling pathway involving interactions between TNF and its receptors, regulating processes such as cell survival, proliferation, inflammation, and apoptosis. It has also indicated that TNF is a crucial pro-inflammatory cytokine in the pathogenesis of rheumatoid arthritis, inhibiting ferroptosis by upregulating solute carrier family 7 member 11 (SLC7A11), glutamate-cysteine ligase modifier subunit (GCLM), and glutamate-cysteine ligase catalytic subunit (GCLC), thereby enhancing cysteine uptake and cellular glutathione (GSH) biosynthesis [53].

According to the Weighted Gene Co-expression Network Analysis (WGCNA) results in Figure 8g, the red module in the CA-L group shows significant upregulation compared to the MOD group. In contrast, the brown module in the CA-M group exhibits significant downregulation. Therefore, visual network node analysis is performed for both modules. The genes with the highest connectivity are interferon regulatory factor 8 (Irf8) and threonine tyrosine kinase (Ttk) (Figure 8h,i). Irf8 is an immunomodulatory gene that regulates immune responses, inflammation, and tumor immunity [54]. Inhibition of Irf8 by CA-M and CA-H may reduce inflammatory infiltration of UVB-induced interferon. Studies have demonstrated that tyrosinase can reduce the inflammatory response to psoriasis and improve symptoms by regulating immune system function. Similarly, Ttk can promote skin cell renewal and reverse skin aging caused by UVB radiation [55].

### 2.5. Metabolic Analysis

Experimental data are imported into MetaboAnalyst 6.0 (https://www.metaboanalyst.ca/ (accessed on 11 January 2025)) for evaluation using principal component analysis (PLS-A) and grouping for differential metabolite screening. Variable importance in projection (VIP) values is calculated using partial least squares regression models to describe the overall contribution of each variable to the model. Typically, VIP values greater than 1 are considered significant differential metabolites. Subsequently, the *t*-test method is used to calculate *p*-values to identify differential metabolites with VIP values greater than 1 and *p*-values less than 0.05. The Venny 2.1 tool (https://bioinfogp.cnb.csic.es/tools/venny/ (accessed on 16 January 2025)) is then used to select common differential metabolites between the treatment group and the model group, as well as between the model group and the control group. Metabolic pathway enrichment is performed using MetaboAnalyst 6.0 and generates a cluster heatmap.

**Figure 9:**
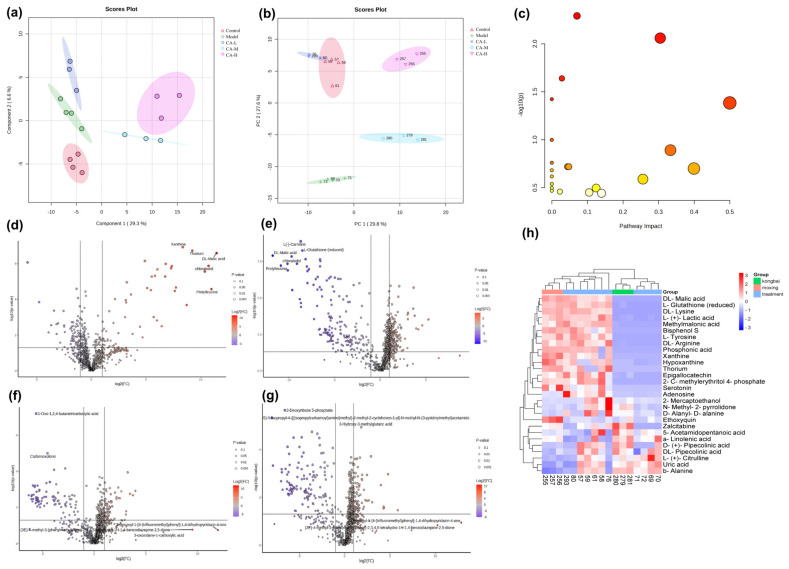
Effects of different concentrations of CA on skin metabolism in UV-induced photoaging mice: (**a**,**b**) PCA and partial least squares (PLS) analyses for metabolic differences among the groups, (**c**) metabolic pathway diagram (Darker red indicates higher statistical significance, and larger diameter represents greater enrichment), (**d**) Control vs. Model, (**e**) Model vs. CA-L, (**f**) Model vs. CA-M, (**g**) Model vs. CA-H, and (**h**) a cluster heat map illustrating the relative levels of various metabolites.

Metabolomic analysis is also performed for all samples. Initially, PCA analysis is performed, followed by PLS-DA analysis that shows improved separation between the control and model groups compared to PCA results. The modeling is shown in Figure 9a,b. Pathway enrichment analysis visualizes metabolic pathways, with 21 pathways collected. CA primarily reduces skin photoaging damage through pathways like ECM-receptor interaction, focal adhesion, the PI3K-Akt signaling pathway, and glycerolipid metabolism. CA improves skin aging by modulating ferroptosis by regulating metabolites such as glutathione, methylmalonic acid, serotonin, and uric acid (Figure 9c). Figure 9d–g shows the volcanic plot of metabolites between the control group and CA upregulated and downregulated photoaging tissues. The cluster heatmap (Figure 9h) indicates that these metabolites are not significantly expressed in the model group but show significant variation after CA treatment, suggesting that CA may modulate metabolic disruptions.

### 2.6. Network Pharmacology Analysis

**Figure 10:**
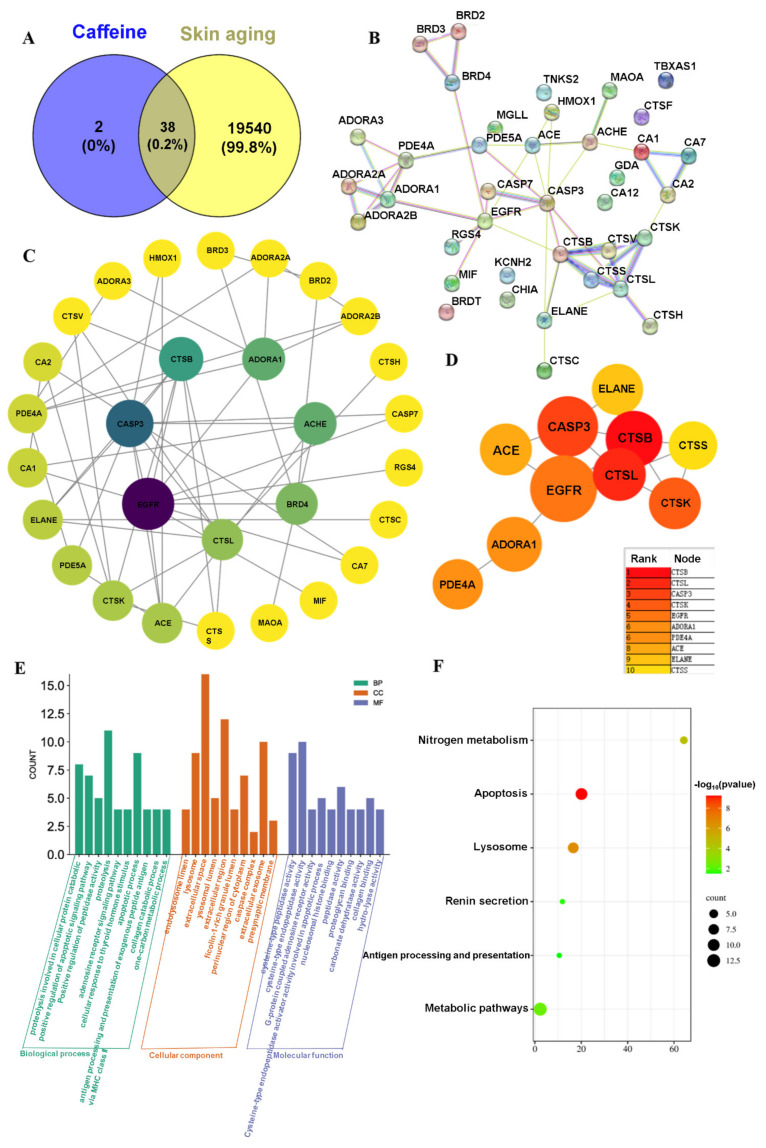
(**A**) CA and skin aging intersection gene Venn diagram: (**B**) protein interaction network diagram, (**C**,**D**) protein interaction network topology map, (**E**) GO enrichment histogram, and (**F**) KEGG histogram.

Using SwissTargetPrediction, 40 potential anti-aging targets for CA are predicted. The GeneCards database is searched using the keyword “skin aging” to identify relevant targets, yielding 19,578 results after removing duplicates. Results are analyzed using an online Venn diagram tool, identifying 38 intersection targets. The resulting Venn diagram illustrates these overlaps (Figure 10A). Moreover, the PPI network for potential CA targets delaying skin photoaging is constructed. Common protein-coding genes are submitted to the STRING database, and a protein–protein interaction (PPI) map is generated (Figure 10B). Subsequently, a compound-target interaction network is constructed based on Cytoscape 3.7.2 software. Core nodes are identified based on network topology features, including node degree values, and the resulting network diagrams are illustrated in Figure 10C,D. In addition, the fundamental function of each gene is determined by its protein domain and the associated research literature. The GO and KEGG databases classify gene functions using distinct classification methods. GO enrichment analysis is conducted using the DAVID database, providing functional annotation tools (ncifcrf.gov). Furthermore, 116 GO entries are identified, including 69 BP, 21 CC, and 26 MF. To understand gene functions, *p*-values and counts are used as reference metrics for sorting, creating a histogram (Figure 10E) with support from the bioinformatics platform (https://www.bioinformatics.com.cn/ (accessed on 25 January 2025)).

Analysis of biological processes reveals that key target genes are primarily engaged in various functions, including proteolysis associated with cellular protein catabolism, positive regulation of the adenosine receptor signaling pathway, apoptotic processes, general proteolysis, apoptotic signaling pathways, cellular responses to thyroid hormone stimuli, positive regulation of peptidase activity, and collagen catabolism. Furthermore, analysis of cellular components suggests associations with endolysosome lumen, lysosome, extracellular space, lysosomal lumen, and caspase complex. Molecular functions identified mainly include cysteine-type endopeptidase activity, cysteine-type peptidase activity, activator activity for cysteine-type endopeptidases involved in apoptotic processes, peptidase activity, collagen binding, and G-protein coupled adenosine receptor activity, among others. In addition, KEGG analysis is performed using the DAVID database, and a total of 6 signaling pathways are identified, among which are the paths through which CA-regulated skin aging, including nitrogen metabolism, apoptosis, lysosomes, renin secretion, antigen processing and presentation, and metabolic pathways (Figure 10F).

### 2.7. Molecular Docking for Predicting Ligand-Protein Interactions

**Figure 11:**
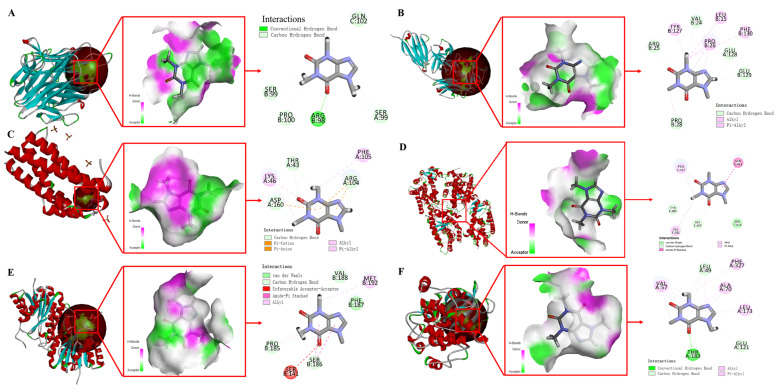
Molecular docking analysis of CA with (**A**) TNF-α (2E7A), (**B**) IL-1β (2E7A), (**C**) IL-6 (1ALU), (**D**) PPAR-α (1K7L), (**E**) NADPH (5B1Y), and (**F**) COL-I (5N3K).

Molecular docking analysis of CA with key proteins, including TNF-α, IL-1β, IL-6, COL-I, NADPH, and PPAR-α, is conducted using the CDOCKER algorithm in Discovery Studio software 2019. From Figure 11, the results show strong binding affinities between CA and target proteins. Specifically, CA exhibits a CDOCKER energy of −27.33 kcal/mol and a CDOCKER interaction energy of −36.3 kcal/mol when docked with TNF-α (Figure 11A). Similarly, docking with IL-1β yields a CDOCKER energy of −20.55 kcal/mol and a CDOCKER interaction energy of −29.73 kcal/mol (Figure 11B). For IL-6, the corresponding values are −15.38 kcal/mol (CDOCKER energy) and −23.31 kcal/mol (CDOCKER interaction energy) (Figure 11C). Docking with COL-I results in a CDOCKER energy of −17.21 kcal/mol and a CDOCKER interaction energy of −25.95 kcal/mol (Figure 11D). Additionally, CA displays a CDOCKER energy of −19.16 kcal/mol and a CDOCKER interaction energy of −27.39 kcal/mol when docked with NADPH (Figure 11E), and values of −27.2 kcal/mol (CDOCKER energy) and −35.6 kcal/mol (CDOCKER interaction energy) when docked with PPAR-α (Figure 11F). According to established criteria, a CDOCKER energy value below −7 kcal/mol indicates a strong binding affinity between the ligand and the protein. From Table 4, CA exhibits significantly low CDOCKER energy values (all below −7 kcal/mol) and high interaction energies with all six proteins, suggesting robust binding stability. These findings imply that TNF-α, IL-1β, IL-6, COL-I, NADPH, and PPAR-α may serve as critical molecular targets through which CA modulates ferroptosis pathways to mitigate skin photoaging. Furthermore, experimental validations have confirmed the presence of robust interactions between CA and these target proteins.

## 3. Discussion

UVB irradiation elicits cutaneous inflammation that constitutes both a causative factor and an exacerbating element in numerous skin disorders. Ferroptosis, a distinct form of programmed cell death tightly associated with aberrant intracellular iron accumulation, is activated in epidermal keratinocytes following UVB exposure. Excess iron catalyzes iron-dependent oxidative stress reactions, thereby inducing lipid peroxidation of cellular membranes and ultimately culminating in cell death [56,57,58,59]. Network pharmacology results indicated that CA regulates skin aging through various metabolic pathways. Previous studies have shown that cell apoptosis is often accompanied by the loss of mitochondrial membrane potential, which further weakens mitochondrial function, leading to energy deficiency and activation of apoptotic signals or promoting ferroptosis. Compared to the UVB group, levels of ferroptosis-related proteins GPX4, ACSL4, FTH1, and NCOA4 are significantly regulated in the CA-treated groups. Integrated analysis of network pharmacology, in vitro experiments, immunohistochemistry, transcriptomics, and metabolomics reveals that the PPAR-α activation mechanism, iron metabolism pathway, oxidative stress, and NF-κB are closely and intricately interconnected, jointly participating in the physiological and pathological regulation of cells. PPAR-α can directly inhibit the activity of NF-κB or indirectly regulate the expression of antioxidant enzymes to reduce oxidative stress levels, thereby suppressing inflammatory responses. Iron metabolism disorders lead to iron overload, which in turn triggers oxidative stress. Moreover, oxidative stress can further affect iron metabolism, and their interaction exacerbates intracellular oxidative damage. Both oxidative stress and iron metabolism disorders can activate the NF-κB signaling pathway through various mechanisms, thereby promoting the production of inflammatory factors and forming a vicious cycle [60,61]. The activation of PPAR-α can indirectly influence the activity of NF-κB by regulating iron metabolism and antioxidant stress, thus alleviating this vicious cycle to a certain extent. This complex interplay plays an important role in the occurrence and development of various diseases, such as chronic inflammatory diseases, iron overload-related diseases, and metabolic diseases, providing potential therapeutic targets for disease treatment. Finally, molecular docking simulation results indicate that CA exhibits a strong spatial binding affinity with several key targets involved in ferroptosis regulation, which may contribute to its protective effects against skin photoaging and other oxidative stress-related conditions.

However, our study focuses on the effects of CA on ferroptosis, but other potential mechanisms through which CA may exert its anti-photoaging effects have not been fully explored. Future research should aim to elucidate these additional mechanisms and validate our findings in human clinical trials to better understand the full potential of CA as an anti-photoaging agent.

## 4. Materials and Methods

### 4.1. Materials

Caffeine (C8H10N4O2, ≥98% purity) was provided by Shenzhen Hujia Technology Co. (Shenzhen, China). Dulbecco’s Modified Eagle Medium (DMEM), phosphate-buffered saline (PBS), and trypsin-EDTA (0.25%) were purchased from Gibco (Grand Island, NE, USA). Fetal Bovine Serum (FBS) was from Vivacell (Shanghai, China). MTT (98%, reagent grade) and 4′,6-diamidino-2-phenylindole (DAPI) solution were purchased from Beyotime (Shanghai, China). The β-galactosidase staining kit and mitochondrial membrane potential detection kit were provided by Addison (Yancheng, China). The DCFH-DA probe was provided by Solarbio (Beijing, China). The SOD activity detection kits, MDA assay kit, and HYP content assay kits were provided by Solarbio (Beijing, China). Bovine serum albumin (BSA), Tween 20 (20 × TBST), Triton X-100, phalloidin and PPAR-α rabbit pAb, goat anti-rabbit IgG second antibody, IL-1β antibody, IL-6 antibody, TNF-α antibody, COL-1 antibody, HRP-labeled second antibody, ACSL4 antibody, GPX4 antibody, FTH1 antibody, and NCOA4 antibody were purchased from Sigma (St. Louis, MO, USA). C11 BODIPY 581/591 probe was purchased from Dojindo (Kyushu, Japan). The FerroOrange probe was from ServiceBio (Wuhan, China).

### 4.2. Cell Viability Testing

Cell survival was determined by tetrazolium salt colorimetry. HaCaT cells (preserved in liquid nitrogen tanks) were regenerated and subcultured (generally through 2–3 generations, which could make cell activity meet the requirements). Inoculated in a 96-well plate, the cell density was 1 × 10^5^ cells per well. Then, 100 μL of culture medium containing CA was added into each well of the healthy plate (the concentration gradient was set to 1.25, 2.5, 5, 10, and 20, a total of five gradients, in μg/mL). The exact amount of aseptic medium was added to the blank well, and the same amount of culture medium containing a specific concentration of cells was added to the control well. After 24 h of culturing, 100 μL of the working solution was added to each well. The 96-well plate was placed in the incubator and left to stand for 4 h. After 4 h, the 96-well plate was removed, the liquid in the 96-well plate was discarded, and 150 μL of dimethyl sulfoxide (DMSO) was added, followed by shaking for 15 min to ensure complete dissolution. The enzyme labelling method measured the Integrated Optical Density (IOD) at 570 nm. The cell survival rate was calculated.

### 4.3. Effects of Caffeine on the Survival Rate of HaCaT Cells Induced by UVB Radiation

Five groups were set up in the cell experiment: the control group, the UVB group, and caffeine-treated groups at high, medium, and low doses (20 μg/mL, 10 μg/mL, and 5 μg/mL). HaCaT cells were cultured for 24 h in 96-well plates, and PBS was used to replace the original culture medium. All groups, except the control group, were exposed to UV radiation at 40 mJ/cm^2^ for modeling purposes. The caffeine treatment groups were cultured using the abovementioned low, medium, and high concentrations. The model and control groups were cultured in a DMEM medium containing FBS only for 24 h, and cell viability was assessed.

### 4.4. Cell Scratch Assay

HaCaT cells were inoculated into the 24-well plate in several 50,000 cells per well. The culture plate was placed in a 37 °C, 5% CO_2_ incubator for cell culture. After 24 h, the supernatant was discarded, and the cells were washed three times with PBS. Complete culture media with different drug concentrations were added, setting up groups as follows: control group, UVB group (40 mJ/cm^2^), CA-L (5 μg/mL) group, CA-M (10 μg/mL) group, and CA-H (20 μg/mL) group. The culture plate was placed in a 37 °C, 5% CO_2_ incubator for cell culture. After replacing the medium with PBS, scratch images at 0 and 24 h were taken under a microscope, with three fields of view selected for each well. The cell migration rate was calculated using Image-Pro Plus 6.0 software, with migration rate = (initial scratch width − post-culture scratch width)/initial scratch width × 100%.

### 4.5. β-Galactosidase Staining

The activity of SA-β-gal in HaCaT cells was detected using a senescence β-galactosidase (SA-β-gal) staining kit. HaCaT cells in the logarithmic growth phase were seeded into a 24-well plate, followed by normal culturing for 24 h before adding the respective drugs. After drug treatment, the culture medium was carefully removed, and each well was washed twice with 1 mL of PBS. Then, 1 mL of β-galactosidase staining fixative solution was added, and the cells were fixed at room temperature for 15 min. After removing the fixative, each well was washed with 1 mL of PBS three times, for 3 min each time. After PBS removal, 1 mL of prepared staining solution was added to each well. Following overnight incubation at 37 °C, SA-β-gal (blue) positive expression in senescent cells was observed and photographed under an optical microscope, and the optical density values of positive areas in the images were analyzed using Image-Pro Plus 6.0 software.

### 4.6. ROS Detection

HaCaT cells were treated under the same conditions for 24 h. The old medium was removed, and 1 mL of complete medium containing different concentrations of CA was added, with the following groups set up: control group (no treatment), UVB group (40 mJ/cm^2^), CA-L (5 μg/mL) group, CA-M (10 μg/mL) group, and CA-H (20 μg/mL) group. After 24 h, the old medium was removed, and the cells were washed three times with PBS. Then, a 10 μM DCFH-DA probe diluted in PBS was added, and the cells were incubated in a 37 °C and 5% CO_2_ incubator for 60 min. Subsequently, the cells were washed three times with PBS for 5 min each time. Images were then observed under an inverted fluorescence microscope in a dark environment with an excitation wavelength of 488 nm, and the optical density values in the images were analyzed using Image-Pro Plus 6.0 software.

### 4.7. Mitochondrial Membrane Potential Level Detection

The 24-well plate with cultured HaCaT cells was removed from a 37 °C, 5% CO_2_ incubator. The culture medium was discarded, and each well was washed with PBS. Then, 1 mL of cell culture medium and 1 mL of pre-prepared JC-1 staining working solution were added, mixed thoroughly, and incubated in a 37 °C and 5% CO_2_ incubator for 20 min. Meanwhile, the JC-1 staining buffer (5×) was diluted fivefold with ultrapure water to prepare 1 × JC-1 staining buffer, which was kept on ice. After incubation, the supernatant was removed, and each well was washed twice with 1 × JC-1 staining buffer, followed by adding 2 mL of cell culture medium. The stained cells were observed under a fluorescence microscope. The collected images were analyzed for fluorescence, and Image Pro Plus 6.0 software was used to calculate the fluorescence intensity of JC-1 aggregates (red) in each group.

### 4.8. Immunofluorescence Analysis

HaCaT cells were inoculated into a 24-well plate at 1 × 10^5^ cells/mL density. After 24 h, the medium was replaced with PBS and subjected to 40 mJ/cm^2^ of UV irradiation. Subsequently, the cells were categorized into control, UVB, and caffeine groups and cultured in incubators. After 24 h, the liquid in the 24-well plate was removed, and the cells were washed three times with PBS for 5 min each. To fix the cells, 1 mL of 4% paraformaldehyde was added at room temperature for 15 min, after which it was discarded, and the cells were washed three times with PBS. 0.5% Triton X-100 was added for 10 min to permeabilize the membranes, after which the 24-well plate was washed three times with PBS. Blocking was conducted using 1 mL of 5% BSA in Tris-buffered saline containing 0.1% 20 × TBST for 1 h. Following removing the blocking solution, 200 µL of PPAR-α primary antibody (1:200, diluted in 1% BSA) was added and incubated overnight at 4 °C, after which the TBST was used to wash the plate three times. Subsequently, 200 µL of goat anti-rabbit fluorescent secondary antibody (1:800, diluted in 1% BSA) was added in the dark for 1 h. Following removing the secondary antibody, the wells were washed three times with TBST. Finally, 200 µL of DAPI staining solution was added for 10 min, followed by three washes with PBS. An inverted fluorescence microscope was used to photograph the cells, and fluorescence intensity values were calculated using Image Pro Plus software.

### 4.9. Cytoskeleton Staining

After 24 h of HaCaT cell culture, the old culture medium was aspirated, and the cells were washed three times with PBS. Subsequently, the cells were fixed with cell fixation solution for 10 min, permeabilized with 0.5% Triton-X100 for 10 min, and washed three times with PBS. TRITC-labeled phalloidin was then added to the dish and incubated at room temperature in the dark for 30 min (TRITC-labeled phalloidin was diluted in 1% BSA to reduce fluorescence background), followed by three washes with PBS. A DAPI solution with a concentration of 10 μg/mL was applied to the dish for dark staining for 10 min, followed by three washes with PBS. An anti-fluorescence quencher was added to the dish, and images were captured under a laser confocal microscope.

### 4.10. Cellular Lipid Peroxide Detection

HaCaT cells were inoculated in 24-well plates. After 24 h of drug treatment, the lipid peroxidation fluorescent probe C11 BODIPY 581/591 working solution was diluted to 50 μmol/L with PBS buffer, and 500 μL of working solution was added to each well of the 24-well plate. The cells were incubated at 37 °C in the dark for 30 min. The cells were washed three times with PBS buffer to remove the fluorescent probe that did not enter the cells. The cells were imaged using an inverted fluorescence microscope. The green and red fluorescence area was calculated using Image Pro Plus software, and each group’s green/red ratio was calculated. A histogram was drawn using GraphPad Prism 6 software.

### 4.11. Intracellular Fe^2+^ Level Detection

HaCaT cells were cultured in 24-well plates and washed three times with PBS buffer 24 h after administration. 5 μmol/L FerroOrange working solution diluted with PBS buffer was added to each well and cultured in a 37 °C, 5% CO_2_ incubator for 30 min. Images were taken under an inverted fluorescence microscope, and fluorescence intensity in the images was analyzed using Image Pro Plus software. Histograms were drawn using GraphPad Prism 6 software, and statistical analysis was performed.

### 4.12. Western Blot

After HacaT cells were modeled and dosed according to the above method, the cells were lysed in an ice bath with PMSF protease inhibitor and RIPA buffer (1:1000) for 20 min. The liquid was centrifuged at 4 °C and 12000 rpm for 10 min. The supernatant was collected and added to the SDS-PAGE Sample Loading Buffer 1X, then denatured by heating at 95 °C for 10 min. Each protein sample of 12 μL was loaded onto the SDS-PAGE for electrophoresis and then moved onto the PVDF membrane. Then, the samples were sealed with 5% skim milk for 1h and incubated at 4 °C overnight with Rabbit polyclonal GPX4 Antibody, ACSL4 Antibody, FTH1 Antibody, and NCOA4 Antibody (Affinity Biosciences Co., Shanghai, China). Goat Anti-Rabbit IgG (H+L) HRP (Affinity Biosciences Co., China) was cleaned and incubated with TBST. Luminescence was performed using an electrochemical luminescence (ECL) kit (BioSharp Co., Hefei, China), and band signals were detected with a fluorescence scanner. The specific band was quantified using ImageProPlus 6.0 software (National Institutes of Health, Bethesda, MD, USA). After data normalization, the histogram was plotted using GraphPad Prism 6 (GraphPad Software, La Jolla, CA, USA).

### 4.13. Animals and Treatment

From a global perspective, countries hold diverse attitudes towards conducting cosmetic testing on animals. The European Union has imposed a comprehensive ban on such testing, emphasizing animal welfare and the utilization of alternatives. Nevertheless, several countries, such as the United States, Japan, and China, still permit cosmetic animal testing under ethical and regulatory guidelines. Despite the availability of in vitro alternatives, limitations hinder comprehensive safety assessments. An increasing number of countries are exploring superior methodologies, aiming to improve in line with international trends in the future. The experimental animals used were SPF-grade KM mice, a total of 30. Each mouse was 5 weeks old and male, and the source of purchase was Guangdong Experimental Animal Center. All procedures involving animal experimentation were reviewed and approved by the Animal Experimental Ethics Committee of the Guangdong University of Technology. The animals were fed at 22 °C for four days before the experiment and randomly divided into different groups, including control, model, CA-L (1 wt% CA dissolved in 0.9% saline), CA-M (2.5 wt% CA dissolved in 0.9% saline), and CA-H (5 wt% CA dissolved in 0.9% saline).

Apart from the control group, the other mice received combined UV radiation once a day (UV = 770 mJ/cm^2^, 30 min/day) for a week. Starting from day 8, the experimental mice received UV radiation once every other day and then had the treatment drugs locally applied once a day to the depilated back area (200 μL per mouse). Following the UV exposure, all mice in the experimental group received their respective treatments. The control group received no treatment, and the UV group had 0.9% saline locally applied. CA-L, CA-M, and CA-H were treated by 200 μL of 1, 2.5, and 5 wt% CA solution, respectively. The photos of the back skin were taken on day 29, and the skin was scored based on the level of inflammation. The scoring criteria were as follows: 1, smooth and without wrinkles or erythema; 2, a small amount of erythema, fine lines, or scabs; 3, slight shallow wrinkles, moderate erythema, or scabbing; 4, a large number of shallow wrinkles, severely distributed erythema, or scabs; 5, skin thickening, deep wrinkles, or skin erosion [16]. Then, the mice were killed by cervical dislocation, and 4% paraformaldehyde was used to soak the skin tissue of the postdeposition area. Skin tissue was stained with HE, TB, Masson, and immunohistochemical staining.

### 4.14. Hematoxylin–Eosin Staining

Skin tissues immersed in 4% paraformaldehyde were fixed for 48 h, then embedded in paraffin and sectioned with a microtome. After dewaxing and hydration, the sections were stained with hematoxylin for 10 min, differentiated in a differentiation solution for 30 s, and stained with eosin for 1 min. This was followed by dehydration, clearing, and mounting. Images were captured using a fully automated digital slide scanning system (Zeiss Axio Scan. Z1, Carl Zeiss AG, Jena, Germany) and analyzed with Image-Pro Plus 6.0 software.

### 4.15. Toluidine Blue Staining

Skin tissue from the lesioned areas on the mice’s backs was immersed in 4% paraformaldehyde. Subsequently, the tissues were embedded in paraffin and sectioned. Following deparaffinization using xylene and dehydration through an ethanol gradient, toluidine blue was used to stain the sections for 2 to 5 min. The sections were rinsed with water, gently differentiated using 0.1% glacial acetic acid, and washed with tap water to halt the reaction while differentiation was monitored under a microscope. The sections were dried in an oven, cleared with xylene for 10 min, and mounted with neutral resin. The scanning system captured images, and mast cell infiltration in the stained sections was analyzed with Image Pro Plus software.

### 4.16. Masson Staining

The paraffin-embedded skin tissue was sequentially immersed in xylene, gradient ethanol, and water. It was then stained with Weigert’s iron hematoxylin for 5 min and then washed with water. Differentiation was performed with 1% hydrochloric acid alcohol for a few seconds, and the sections were rinsed with running water. Next, the tissue was stained with Ponceau acidic fuchsin solution for 5 min, washed with water, and stained with phosphomolybdic acid aqueous solution for another 5 min. This was followed by counterstaining with aniline blue solution for 5 min and differentiation with 1% glacial acetic acid for 1 min. The sections were then dehydrated using gradient ethanol and mounted with neutral resin. Images were captured using an automated slide scanning system, and the optical density values in the images were analyzed using Image-Pro Plus software.

### 4.17. Analysis of SOD, HYP, and MDA

An appropriate amount of skin tissue was taken from storage at −80 °C and subjected to freeze-thaw cycles on ice. For each 100 mg of skin tissue, 1 g of pre-cooled PBS was added, and the tissue was processed using a tissue homogenizer until a homogeneous solution was obtained, resulting in a 10% (*w*/*v*) skin tissue homogenate. The high-speed centrifuge was adjusted to 4 °C and operated at 10,000 r/min for 10 min, repeating this step twice to collect the skin supernatant, which was reserved for further use. Before testing, all samples were standardized for protein concentration using the bicinchoninic acid (BCA) method. The contents of SOD, HYP, and MDA were measured according to the instructions provided in the respective assay kits. Finally, data analysis was performed using GraphPad Prism software.

### 4.18. Immunohistochemistry Analysis

The paraffin sections underwent deparaffinization with xylene and were dehydrated through a gradient of anhydrous ethanol. PBS was used to wash the sections after incubating with 3% hydrogen peroxide for 10 min. After serum blocking, the sections were incubated overnight at 4 °C with primary antibodies targeting TNF-α, IL-6, IL-1β, and COL-I, and then incubated at 37 °C with HRP-labeled anti-rabbit IgG. After this, the sections were stained with diaminobenzidine (DAB) and counterstained with hematoxylin. After mounting, images were obtained by the scanning system, and the average optical density was analyzed.

### 4.19. Transcriptome Sequencing and Analysis

The completed transcriptome processing workflow was as follows: the mouse skin samples were taken out from −80 °C and transported to Shanghai Majorbio Bio-pharm Technology Co., Ltd. (Shanghai, China). Based on the Illumina NovaSeq 6000 sequencing platform, high-throughput sequencing was performed on all mRNA transcribed from skin tissues. The specific experimental process was as follows: total RNA was extracted first, and then mRNA was specifically enriched with Oligo dT; next, the enriched mRNA was fragmented to meet the requirements for subsequent sequencing; subsequently, cDNA was synthesized by reverse transcription using the fragmented mRNA as the template; the synthesized cDNA ends were ligated with specific adaptor sequences to facilitate recognition and positioning on the sequencing platform; finally, the completed sequencing library was loaded onto the Illumina sequencer, the sequencing program was initiated, and high-precision sequencing data were obtained. To perform transcriptome sequencing of library data, this paper used the Illumina HiSeq4000 platform for analysis. Low-quality data were filtered out of the original by a fast quality control method to ensure data quality. This process involved removing sequences that contained linkers, had more than 10% uncertain bases (N), included base A, or contained more than 50% low-quality bases (Q ≤ 20). Ribosomal alignment, sequencing alignment, and reference genome alignment were performed, followed by transcript reconstruction to calculate gene levels in the samples. Sample correlation and differential expression analyses were conducted, with genes marked significantly differently if their FDR was <0 and |log_2_(FC)| > 1. These analyses used short sequence alignment tools such as Bowtie2, HISAT2, StringTie, R, and DESeq. The differentially expressed proteins were compared and tested one by one in various terms in the GO database, and the differentially expressed genes were condensed and analyzed by GO. In addition, the sequencing results were compared with the KEGG database, and the differentially expressed genes were functionally annotated and classified.

### 4.20. Metabolomics Analysis

The selected metabolomics method was based on earlier studies. A total of 0.1 g of skin tissue was mixed with 1 mL of chromatography-grade methanol and then homogenized by centrifugation at 13,000 rpm for 15 min at 4 °C. The supernatant was subsequently filtered using a 0.22 μm filter (NEST Biotechnology (Wuxi, China)), and 100 μL of the filtrate was transferred into vials for further analysis. Chromatographic separation was conducted using an ultrahigh-performance liquid chromatography (UPLC) system. The hydrophilic interaction liquid chromatography column was maintained at 25 °C with 0.5 mL/min, and 2 μL of liquid was injected into it. The mobile phase consisted of A: water combined with 25 mmol/L ammonium acetate and 25 mmol/L ammonia, and B: acetonitrile. The elution conditions were as follows: 0 to 0.5 min at 95% B; 0.5 to 7 min from 95% to 65% B; 7 to 8 min from 65% to 40% B; 8 to 9 min at 40% B; 9 to 9.1 min from 40% to 95% B; and 9.1 to 12 min at 95% B. To collect samples, the AB Triple TOF 6600 mass spectrometer was employed to acquire primary and secondary spectra. The ESI source conditions consisted of ion source gas ratios of 1:60 and 2:60, with a current gas flow of 30. The ion spray floating voltage was set to ±5500 V, operating in both positive and negative modes. TOF scanning was performed in the *m*/*z* range of 60–1000 Da, and production scanning was 25–1000 Da. The collected data were analyzed by secondary mass spectrometry in high sensitivity mode, with clustering potentials of ±60 V for both modes and collision energy of (35 ± 15) eV.

### 4.21. Network Pharmacology Analysis

A search for “skin aging” in the GeneCards database (www.genecards.org/, (accessed on 23 July 2025)) yielded targets associated with skin aging. The potential targets of differential metabolites (DMs) were extracted from the SEA (https://sea.bkslab.org/, (accessed on 23 July 2025)) and TCMSP databases (https://www.tcmsp-e.com/load_intro.php?id=43 (accessed on 23 July 2025)), and their intersections with skin aging were analyzed using Venny (https://bioinfogp.cnb.csic.es/tools/venny/index.html, (accessed on 23 July 2025)). The STRING tool (www.string-db.org/, (accessed on 23 July 2025)) was employed to investigate direct and indirect interactions among these targets. Subsequently, a preliminary PPI network map was constructed to depict the top 20 key targets. The DAVID tool (https://david.ncifcrf.gov, (accessed on 23 July 2025)) was used to categorize GO terms and enrich pathways in the KEGG. The visualization of GO terms and KEGG pathways was achieved using the online platform Weishengxin (https://www.bioinformatics.com.cn/ (accessed on 23 July 2025)).

### 4.22. Molecular Docking

The three-dimensional structures of small-molecule ligands were obtained from PubChem (https://pubchem.ncbi.nlm.nih.gov/, (accessed on 23 July 2025)), while protein structures were retrieved from the PDB database (RCSB PDB: Homepage) (TNF-α: 2E7A; IL-1β: 2E7A; IL-6: 1ALU; COL-I: 5N3K; NADPH: 5B1Y; PPARα: 1K7L). The ligand was optimized using the Prepare Ligand module in Discovery Studio (version 19.1.0) with the default settings. The CDOCKER algorithm was used to determine the proteins’ active sites, employing default settings. A total of 10 runs were conducted, and the optimal binding pose for each ligand was selected according to the CDOCKER interaction energy.

### 4.23. Statistical Analysis

The data were presented as the mean ± standard error. Statistical analysis was performed using GraphPad Prism software, version 8.0.2. One-way analysis of variance (ANOVA) was used to compare the means across multiple groups, while pairwise comparisons between smaller groups were performed using a two-sided Student’s *t*-test. A *p*-value of less than 0.05 was deemed statistically significant, while *p*-values below 0.01 indicated a substantial difference.

## 5. Conclusions

In summary, this study indicates that caffeine (CA) can improve UV-induced skin aging and damage by inhibiting the ferroptosis pathway. CA reduces β-galactosidase activity in photoaged HaCaT cells, promotes cell proliferation, and alleviates cell senescence. CA also significantly reduces ROS levels in photo-aged cells and increases mitochondrial membrane potential, effectively inhibiting ferroptosis. In addition, CA enhances the expression of PPAR-α in photoaged cells. It repairs the skeletal structure of HaCaT cells after UV irradiation, further demonstrating the repair effect on UV-induced cell damage. CA also inhibits UV-induced epidermal hyperplasia in mouse skin, reduces mast cell infiltration, promotes collagen fiber repair, downregulates the expression of pro-inflammatory factors (such as IL-6, TNF-α, and IL-1β), upregulates the expression of key collagen COL-1, and further reduces the aging effects. It also regulates UV-induced oxidative stress by downregulating HYP and MDA levels and upregulating SOD levels in the skin. Comprehensive analyses of transcriptomics, metabolomics, and network pharmacology show that CA can indirectly affect ferroptosis by regulating immune response, cell apoptosis, and inflammation-related metabolic pathways, thereby alleviating photoaging damage. Molecular docking confirms the strong interaction of CA with key ferroptosis-related targets, including IL-6, IL-1β, TNF-α, COL-I, PPAR-α, and NADPH. Further studies indicate that CA reduces UV-induced lipid peroxidation in HaCaT cells, lowers intracellular Fe^2+^ levels, and regulates the expression of key ferroptosis-related proteins such as GPX4, ACSL4, FTH1, and NCOA4, demonstrating its potential as an anti-photoaging agent.

## Figures and Tables

**Table 1:** Comparison of changes in enzyme content in mice skin tissue.

Group	SOD/(U·Mgprot^−1^)	HYP/(μg·Mgprot^−1^)	MDA/(nmol·Mgprot^−1^)
Control	223.87	8.22	2.16
UV	56.88	11.38	6.90
CA-L	88.05	10.66	6.10
CA-M	100.46	9.82	5.04
CA-H	122.43	8.53	4.62

**Table 2:** Common cell senescence-related genes and gene regulation.

Gene ID	Gene	Gene Description	*p*-Value			Regulatory Situation
			CA-L	CA-M	CA-H	
**ENSMUSG00000009185**	**Ccl8**	**Chemokine (C-C motif) ligand 8**	3.54 × 10^2^	9.38 × 10^4^	-	Down
**ENSMUSG00000020427**	**Igfbp3**	**Insulin-like growth factor binding protein 3**	4.53 × 10^2^	2.46 × 10^2^	-	Up
**ENSMUSG00000034855**	**Cxcl10**	**Chemokine (C-X-C motif) ligand 10**	1.95 × 10^4^	3.40 × 10^3^	2.24 × 10^3^	Down
**ENSMUSG00000035042**	**Ccl5**	**Chemokine (C-C motif) ligand 5**	3.32 × 10^4^	8.50 × 10^3^	1.11 × 10^2^	Down
**ENSMUSG00000037225**	**Fgf2**	**Fibroblast growth factor 2**	1.07 × 10^2^	4.08 × 10^2^	1.41 × 10^3^	Up
**ENSMUSG00000049723**	**Mmp12**	**Matrix metallopeptidase 12**	4.31 × 10^3^	1.07 × 10^6^	1.23 × 10^3^	Down

**Table 3:** KEGG pathways and related genes.

Pathway Description	*p* Value	Gene Name	First Category	Second Category
Circadian rhythm	1.06 × 10^6^	Nr1d1, Nr1d2, Npas2, Dbp	Organismal systems	Environmental adaptation
α-Linolenic acid metabolism	1.51 × 10^3^	Pla2g4c, Pla2g2d	Metabolism	Lipid metabolism
PPAR signaling pathway	3.91 × 10^6^	Angptl4, Plin4, Plin5, Sorbs1, Pck1	Organismal systems	Endocrine system
Glycolysis/gluconeogenesis	6.77 × 10^4^	Fbp2, Sorbs1, Pck1, Gck	Metabolism	Carbohydrate metabolism
Insulin signaling pathway	4.81 × 10^4^	Fbp2, Pck1, Gck	Organismal systems	Endocrine system
Glucagon signaling pathway	2.67 × 10^3^	Fbp2, Pck1, Gck	Organismal systems	Endocrine system
Chemokine signaling pathway	7.07 × 10^4^	Hck, Cxcl9, Ccl12	Organismal systems	Immune system
Viral protein interaction with cytokine and cytokine receptor	3.45 × 10^3^	Cxcl9, Ccl12	Environmental information processing	Signaling molecules and interaction

**Table 4:** Molecular docking results of CA with six proteins.

Receptor	CDOCKER Energy (kcal/mol)	CDOCKER Interaction Energy (kcal/mol)
TNF-α (PDB: 2E7A)	−27.33	−36.3
IL-1β (PDB: 2E7A)	−20.55	−29.73
IL-6 (PDB: 1ALU)	−15.38	−23.31
COL-I (PDB: 5N3K)	−17.21	−25.95
NADPH (PDB: 5B1Y)	−19.16	−27.39
PPAR-α (PDB: 1K7L)	−27.2	−35.6

## Data Availability

The original contributions presented in this study are included in the article. Further inquiries can be directed to the corresponding author.
